# The prevalence of cervical cytology abnormalities and human papillomavirus in women infected with the human immunodeficiency virus

**DOI:** 10.1186/1750-9378-4-S1-S8

**Published:** 2009-02-10

**Authors:** Dionne N Dames, Camille Ragin, Andrea Griffith-Bowe, Perry Gomez, Raleigh Butler

**Affiliations:** 1 Department of Medicine, Princess Margaret Hospital, Nassau, Bahamas; 2 Department of Obstetrics & Gynaecology, Princess Margaret Hospital, Nassau, Bahamas; 3 Department of Epidemiology, Graduate School of Public Health, University of Pittsburgh, Pittsburgh, USA; 4 Division of Cancer Prevention and Population Science, University of Pittsburgh Cancer Institute, Pittsburgh, USA; 5 Department of Epidemiology and Biostatistics, Downstate School of Public Health, State University of New York, USA

## Abstract

**Introduction:**

The human papillomavirus (HPV) is the major etiologic agent in the development of cervical cancer and its natural history of infection is altered in persons infected with the human immunodeficiency virus (HIV). The prevalence of HPV infection and cervical dysplasia in the HIV sero-positive females in the Bahamas is not known. Finding out the prevalence would allow for the establishment of protocols to optimize total care of this population and help prevent morbidity and mortality related to cervical cancer.

**Objective:**

The Objective of this study is to determine the prevalence of high risk HPV genotypes and the prevalence of cervical dysplasia in the HIV sero-positive females attending the Infectious Disease Clinic at the Princess Margaret Hospital, Nassau, Bahamas.

**Methods:**

One hundred consecutive, consenting, non-pregnant, HIV-sero-positive females from the Infectious Disease Clinic at the Princess Margaret Hospital in Nassau, Bahamas were screened for high-risk HPV infections and cervical cytology abnormalities using liquid-based pap smear and signal amplification nucleic acid method for HPV detection. A questionnaire was also utilized to gather demographic information and obtain information on known risk factors associated with HPV infections such numbers of partners.

**Results:**

The prevalence of high-risk HPV was 67% and cervical abnormalities were noted in 44% of the study population. High-risk HPV types were more likely to be present in women with CD4+ cell counts less than 400 μl^-1 ^and in women with cervical cytology abnormalities (97%). The most common cervical abnormality was low-grade squamous intraepithelial lesions.

**Conclusion:**

Findings suggest that HIV-sero positive females should have HPV testing done as part of their normal gynecology evaluation and these patients should be encouraged and provisions be made for ease of access having regular PAP smears and HPV testing.

## Background

The human papillomavirus (HPV) is the major etiologic agent in the development of cervical cancer [1] with known risk factors such an early age at first intercourse, history of multiple sexual partners, oral contraceptive use, high parity, lower socioeconomic status, and immunosuppression. In immunocompetent subjects, HPV infections normally clear in six to twenty-four months in 70% of females [2]. The natural history of HPV infection is altered in persons infected with the human immunodeficiency virus (HIV) and there is an increased likelihood of persistent HPV infections in this population [3]. This persistent infection increases their risk of having cervical dysplasia and cervical intraepithelial neoplasms (CIN) [3,4]. HIV-positive women especially those with severe immunosuppression are five times more likely than HIV sero-negative women to have lower genital tract neoplasias [4,7]. High HPV load in HIV-positive women is associated with a 10-fold increase risk of CIN in severe immunosuppression [5]. Recent studies indicate that plasma HIV RNA levels and CD4+ cell counts are strong determinants of the ability to detect HPV suggesting that as the immune system weakens, it facilitates reactivation of the HPV thus helping to explain the increase rates of HPV infection in women with HIV [6,7]. Additionally, Highly Active Anti-Retroviral Therapy (HAART) does not seem to impact this increased rate or persistent of HPV infections in this population [8]. For many years the focus of care for HIV sero-positive patients in the Bahamas was on immune system reconstitution using HAART. Patients receiving HAART die less frequently from opportunistic infections but more now face morbidity from chronic medical diseases and other illnesses including malignancies. One such preventable disease is cervical cancer. There is no available data from the Bahamas or the Caribbean that documents the prevalence of cervical dysplasia or HPV infections in HIV sero-positive females.

### Study goal

The goal of the study is to determine the prevalence of high-risk HPV infections and associated cervical abnormalities in HIV sero-positive females in the Bahamas.

## Methods

### Study population

One hundred (100) consecutive, consenting, non-pregnant, HIV sero-positive females (18) years or older were recruited from the Infectious Disease Clinic at the Princess Margaret Hospital in Nassau, Bahamas. The criteria for eligibility required all of the following: Females 18 years and older, a positive HIV via ELISA and Western Blot methods and HIV infectivity via sexual intercourse. All participants were interviewed, provided informed written consent and had a liquid based pap smeared performed after receiving consent. The study received ethical approval from the Ethics Committee of the Public Hospital Authority and the University of the West Indies – Bahamas Campus.

### Sample and data collection

Each participant had a vaginal examination performed by an Obstetrics and Gynaecology Resident. Cervical samples were collected using a cytobrush (cytobroom) and stored in Sure Path vials. A cytotechnologist screened the prepared slides. All positive results and fifteen percent (15%) of all negatives results were reviewed by a board certified cytopathologist. The cytology was reported as either negative, atypical squamous cells of unknown significance (ASCUS), low-grade squamous intraepithelial lesion (LGSIL) or high-grade squamous intraepithelial lesion (HGSIL). Additionally co-infections as well as presence of HPV changes were noted. The Obstetrics and Gynaecology Oncologist referred all patients with abnormal cytology results to Colposcopy Clinic for further evaluation, treatment and follow-up as determined.

Each patient was interviewed and a questionnaire completed by the interviewer. Information on associated HPV risk factors such as parity, number of sexual partners, and birth control method, risky behavior such as drug use, sex under the influence of drugs and condom usage as well as demographic were obtained via the questionnaire. The medical records were reviewed to obtain patient's date of HIV testing and confirm sero-positivity, most recent CD4+ lymphocyte counts and viral load, and use of high active anti-retroviral therapy.

### CD4+ and viral load

Samples collected from patients were stained with FlowCARE PLG CD4 Reagent. CD4+ lymphocytes counts were determined by flow cytometry. White blood cell counts were obtained with a COULTER STKS Instrument. The values were expressed in terms of percentage (%) of total lymphocytes and absolute count (cells/μL).

The DNA HIV viral load was tested using a quantiplex HIV-1 RNA 3.0 Assay. This assay is a nucleic acid hydridization procedure to directly quantify HIV-1 RNA in human plasma. The HIV-1 is concentrated from the plasma by centrifugation then the genomic RRNA is liberated from the virions and captured on a microwell by specific synthetic oligonucleotide capture probes. The probes bind to different regions of the pol gene of the viral RNA which is them amplified.

Multiple copies of the alkaline phosphatase-labelled probe are then hybridized to an immobilized complex to amplify the signal. Detection is achieved by incubating the complex with a chemiluminescent substrate. The light emission generated by the bound alkaline phosphatase reacting with the chemiluminescent substrate is recorded as relative light units (RLUs). The light emission is directly proportional to the amount of HIV-1 present in each sample. A standard curve is defined by light emission from standards containing known concentrations of beta proiolactone (BPL)-treated virus. Concentrations of HIV-1 RNA in plasma specimens are determined form this standard curve.

### HPV testing

HPV was detected using the HPV Hybrid Capture II assay which is a signal amplification nucleic acid method for the qualitative detection of eighteen types of human papillomavirus (HPV) DNA in cervical specimens. The assay differentiates between high risk and low risk groups. High HPV types detected are 16, 18, 31, 33, 35, 39, 45, 51, 52, 56, 58, 59 and 68.

### Statistical analysis

Clinical and laboratory risk factors for the study population were imported to a statistical software package for analysis. Statistical analyses were performed using the Intercooled STATA (version SE 10.0) software (StataCorp. LP, College Station TX). Factors associated with high-risk HPV status were selected based on cross-tabulations and univariate logistic regression analyses. Cross-tabulations were analyzed using the Fisher's exact test. Logistic regression was used to perform the analyses of associations between demographic, behavioral and clinical variables with high-risk HPV status. Crude odds ratios were first calculated. Potential confounders were identified based on p-values = 0.05. Logistic regression was used to calculate the relative odds of high-risk HPV status after adjusting for the potential confounders (age (continuous), marital status, number of pregnancies, Tobacco use, duration of HAART therapy, CD4 counts, viral load and cervical cytology).

## Results

### Characteristics of the study population

One hundred females ranging from ages 20–71 years were interviewed and had a PAP smear performed. The mean and median ages were 40.1 years and 39 years respectively. Fifty-six participants (56%) were single and twenty-four (24%) were married (Table 1). Ninety-four participants were Bahamians with only 6% accounting for other nationalities. Thirty-nine percent reported their age of first sexual intercourse prior to age 16 years and seventy participants (70%) were sexually active before the age of 18 years. Sixty participants (60%) had five or more lifetime sexual partners.

**Table 1:** Demographics and characteristics of the study population

	***N***
Marital Status	
Single	56
Married	24
Divorced	9
Widowed	11
Nationality	
Bahamians	94
Haitians	4
Jamaicans	2
Alcohol Use	
Never	52
Ever	48
Marijuana Use	
Never	82
Ever	18
Tobacco Use	
Never	89
Ever	11
HAART Therapy	
No	18
Yes	81
Cervical Cytology	
Negative	56
ASCUS	7
LGSIL	31
HGSIL	6
High Risk HPV	
Negative	32
Positive	67
Not available	1
Total	100

Sixty-seven women (67%) tested positive for high-risk HPV genotypes. The proportion of high-risk HPV-positive women was significantly higher than those who are HPV-negative (p = 0.001). Forty-four women (44%) were found to have abnormal cervical cytology with 95% testing positive for high-risk HPV. Seven patients (7%) had ASCUS, LGSIL in 31 (31%) and HGSIL in six (6%) participants.

### High-risk HPV infections and behavior

Adjusting for potential confounders (Adjusted Odds Ratio [AdjOR] 27.0, 95% confidence interval [CI] 2.0–355.3), one hundred percent of participants with HGSIL were high-risk HPV positive (Table 2).

**Table 2:** Association of high-risk HPV with behavioral variables

	High Risk HPV Infections			
	Negative (32) N	Positive (67) N	Odds Ratio (95% CI)	Adjusted Odds Ratio (95% CI)*
Age				
<40	10	42	1.0 (ref)	1 (ref)
40+	22	25	0.3 (0.1–0.7)	0.4 (0.1–1.5)
Birth-control method				
None	5	7	0.6 (0.1 – 2.3)	0.3 (0.0 – 5.1)
Barrier	10	24	1.0 (ref)	1.0 (ref)
No barrier	2	12	2.5 (0.5–13.3)	6.8 (0.7 – 67.8)
Abstinence	15	24	0.7 (0.3 – 1.8)	0.5 (0.1 – 2.5)
Age at menarche				
<13 years	10	22	1.0 (ref)	1.0 (ref)
13+ years	22	45	0.9 (0.4 – 2.3)	2.3 (0.5–9.8)
Number of pregnancies				
<3	6	31	1.0 (ref)	1.0 (ref)
3+	26	36	0.3 (0.1 – 0.7)	0.2 (0.1 – 0.99)
Age of first sexual intercourse				
<16 years	12	27	1.0 (ref)	1.0 (ref)
16+ years	20	40	0.9 (0.4 – 2.1)	1.0 (0.3 – 3.8)
Lifetime number of sex partners				
<5	11	28	1.0 (ref)	1.0 (ref)
5 to 10	16	24	0.6 (0.2 – 1.5)	1.2 (0.3 – 5.4)
>10	5	15	1.2 (0.3 – 4.0)	4.4 (0.7 – 29.6)
Alcohol				
Never	18	34	1.0 (ref)	1.0 (ref)
Ever	14	33	1.3 (0.5 – 2.9)	1.7 (0.4 – 6.8)
Marijuana				
Never	27	54	1.0 (ref)	1.0 (ref)
Ever	5	13	1.3 (0.4 – 4.0)	7.0 (0.5 – 91.2)
Tobacco				
Never	27	63	1.0 (ref)	1.0 (ref)
Ever	7	4	0.2 (0.1 – 0.8)	0.2 (0.04 – 1.6)

ref = reference; *Adjusted for age (continuous), marital status, number of pregnancies, Tobacco use, duration of HAART therapy, CD4 counts, viral load and cervical cytology; Birth control method: Barrier = male condom, No barrier = birth control pill or Depo-Provera^®^

There were no statistically significant associations observed between high-risk HPV infections and alcohol use, marijuana use, tobacco use or cocaine use. There were no associations between high-risk HPV infections and genital herpes, gonorrhea, syphilis, chancroid, trichomoniasis or Chlamydia infections, (p > 0.1). Of note, participants with greater than ten lifetime sexual partners were four times more likely to have high-risk HPV infections than those with less than 10 lifetime sexual partners (AdjOR 4.4, 95% CI 0.7 – 29.6). This finding was not statistically significant.

### High-risk HPV infection and clinical correlates of HIV infection

The mean and median CD4+ lymphocyte counts were 339.15 and 260 respectively. Twenty-three participants (23%) had CD4+ counts ≥ 500 μl^-1^, 38 (38%) had CD4+ counts between 200 and 499 μl^-1^, and 39 (39%) had CD4+ counts less that 200 μl^-1^. After adjusting for potential confounders, subjects with CD4+ counts greater than 400 μl^-1 ^were less likely to test positive for high-risk HPV (AdjOR 0.2, 95% CI: 0.05 – 0.8, p = 0.019) (Table 3).

**Table 3:** Association of high-risk HPV infection with variables associated with HIV infection

	High Risk HPV Infections			
	Negative (32) N	Positive (67) N	Odds Ratio (95% CI)	Adjusted Odds Ratio (95% CI)*
Years living with HIV				
<2	8	21	1 (ref)	1 (ref)
2 to 6)	12	13	2.4 (0.8 – 7.5)	3.0 (0.5 – 18.5)
6+	12	33	2.5 (0.9 – 7.1)	1.7 (0.3 – 9.2)
Duration of HAART therapy				
<3	12	42	1 (ref)	1 (ref)
3+	20	25	0.4 (0.1 – 0.9)	0.4 (0.1 – 1.4)
CD4 count				
<400	11	57	1 (ref)	1 (ref)
400+	21	10	0.1 (0.03 – 0.2)	0.2 (0.05 – 0.8)
HIV viral load				
<50	23	22	1 (ref)	1 (ref)
50 – 28,000	8	26	3.4 (1.3 – 9.1)	1.9 (0.5 – 7.2)
>28,000	1	19	19.9 (2.4 – 161.3)	2.2 (0.2 – 25.9)
Cervical cytology				
Negative	29	25	1 (ref)	1 (ref)
ASCUS	1	6		
LGSIL	2	29		
HGSIL	0	6		

ref = reference; *Adjusted for age (continuous), marital status, number of pregnancies, Tobacco use, duration of HAART therapy, CD4 counts, viral load and cervical cytology

## Discussion

High-risk HPV is prevalent in the HIV sero-positive females attending the Infectious Disease Clinic at the Princess Margaret Hospital. Unfortunately, a comparison with the HIV sero-negative population could not be done as there is no published HPV prevalence study for the general population. The high prevalence reported in this study is not unusual with HPV infections being quoted as anywhere from 33–47% in other published studies [9-12]. The increased risk of HPV infections in sero-positive women with multiple partners and early onset of sexual activities only confirm what multiple studies have demonstrated previously. There is a strong correlation between the presence of high-risk HPV and cervical cytology abnormalities. Forty-one of the forty-four patients with cervical cytology abnormalities were high-risk HPV positive demonstrating the role of high-risk HPV in cervical dysplasia. Participants were not tested for low-risk HPV genotypes. HIV sero-positive women tend to be infected with multiple genotypes of HPV [10,11]. Three participants with cervical abnormalities were not high-risk HPV positive and may have infections with low risk HPV genotypes. There is a lack of evidence to suggest that low-risk HPV genotypes do not play a role in the increase level of cervical abnormalities and cervical intraepithelial lesions in this immune-compromised population.

Subjects living with HIV infection for two to six years were three times more likely to carry high-risk HPV infections than those infected for less than two years, however this association was not significant (p > 0.1). Similarly, women who lived with HIV infections for more than 6 years were almost two times more likely to carry high-risk HPV infections however; this also was not significant.

Individuals with a CD4+ lymphocyte count of less than 400 μl-^1 ^were more likely to test positive for high-risk HPV genotypes. As CD4+ count is a reflection of the state of the immune system, it reasonable to assume that the immune dysfunction noted at lower CD4+ levels is responsible for the increased detection of high-risk HPV in these women.

An increased number of lifetime sexual partners was associated with an increased likelihood of testing positive for high-risk HPV. Other behavioral variables such as cocaine use, alcohol use and non-barrier method did not show the expected increased risk. This lack of increased risk could be the result of a relatively small study population.

## Conclusion

The high prevalence of high-risk HPV genotypes and cervical cytology abnormalities demonstrates the need to screen and monitor HIV sero-positive women and to implement preventative measures to decrease the numbers of females with high risk HPV infections. Most participants reported not having yearly PAP smears and unfortunately, routine HPV screening is not conducted at the public facilities where most of these patients receive their PAP smears. Screening for HPV infections in HIV sero-positive females should be an integral component of their routine gynaecological evaluation and these patients should be encouraged and provisions be made for ease of access to having regular PAP smears and HPV testing.

## Competing interests

The authors declare that they have no competing interests.

## Authors' contributions

DND conceived of the study, participated in its design and coordination and drafted the manuscript. CR performed the statistical analysis and reviewed the manuscript. AGB performed the Pap smears and cervical examination. RB sourced the funding, helped with coordination of the study, provided further management for patients with detected abnormalities and reviewed the manuscript.
